# Genome of a novel *Sediminibacterium* discovered in association with two species of freshwater cyanobacteria from streams in Southern California

**DOI:** 10.1093/g3journal/jkac123

**Published:** 2022-05-26

**Authors:** Arun Sethuraman, Rosalina Stancheva, Ciara Sanders, Lakme Caceres, David Castro, Hannah Hausknecht-Buss, Simone Henry, Haven Johansen, Antolette Kasler, Sandy Lastor, Isabelle Massaro, Immanuel Mekuria, Andrea Moron-Solano, Niki Read, Gretchen Vengerova, Andrew Zhang, Xiaoyu Zhang, Betsy Read

**Affiliations:** Department of Biology, San Diego State University, San Diego, CA 92182, USA; Department of Biological Sciences, California State University San Marcos, San Marcos, California USA 92096, USA; Department of Biology, San Diego State University, San Diego, CA 92182, USA; Department of Biology, San Diego State University, San Diego, CA 92182, USA; Department of Biology, San Diego State University, San Diego, CA 92182, USA; Palomar College, San Marcos, CA 92069, USA; University of San Diego, San Diego, CA 92110, USA; Department of Biology, San Diego State University, San Diego, CA 92182, USA; Department of Biology, San Diego State University, San Diego, CA 92182, USA; Scripps College, Claremont, CA 91711, USA; Department of Biology, San Diego State University, San Diego, CA 92182, USA; Scripps College, Claremont, CA 91711, USA; Department of Biology, San Diego State University, San Diego, CA 92182, USA; Western Washington University, Bellingham, WA 98225, USA; Department of Biology, San Diego State University, San Diego, CA 92182, USA; Department of Biology, San Diego State University, San Diego, CA 92182, USA; Bay Path University, Long Meadow, MA 01106, USA; Department of Biology, San Diego State University, San Diego, CA 92182, USA; Department of Biology, San Diego State University, San Diego, CA 92182, USA; Department of Biology, San Diego State University, San Diego, CA 92182, USA; University of California, Davis, Davis, CA 95616, USA; Department of Biology, San Diego State University, San Diego, CA 92182, USA; Department of Biology, San Diego State University, San Diego, CA 92182, USA; City College of San Francisco, San Francisco, CA 94112, USA; Department of Biology, San Diego State University, San Diego, CA 92182, USA; Indiana University Bloomington, Bloomington, IN 47405, USA; Department of Biology, San Diego State University, San Diego, CA 92182, USA; Department of Biology, San Diego State University, San Diego, CA 92182, USA

**Keywords:** genome assembly, sediminibacteria, annotation, phylogenomics

## Abstract

Here, we report the discovery of a novel *Sediminibacterium* sequenced from laboratory cultures of freshwater stream cyanobacteria from sites in Southern California, grown in BG11 medium. Our genome-wide analyses reveal a highly contiguous and complete genome (97% BUSCO) that is placed within sediminibacterial clades in phylogenomic analyses. Functional annotation indicates the presence of genes that could be involved in mutualistic/commensal relationship with associated cyanobacterial hosts.

## Introduction

The genus *Sediminibacterium*, first described by Qu and Yuan (2008), is a member of the Chitinophagaceae family within the Bacteroidetes phylum. These gram-negative bacteria that are most closely related phylogenetically to Terrimonas and Niabella (Qu and Yuan 2008) are actively mobile by gliding and can be strict or facultative anaerobes, or obligate aerobes (Kim *et al.* 2013). Currently, 8 species of *Sediminibacterium* have been isolated from sediments derived from freshwater reservoirs, sewage, activate sludge, soil from ginseng fields, and fishbowls (Ayarza *et al.* 2014; Kang *et al.* 2014; Kim *et al.* 2013, 2016; Song *et al.* 2017; García-López *et al.* 2019; Wu *et al.* 2021). During the study of 2 freshwater cyanobacteria (blue-green algae), the genome of a *Sediminibacterium* resident in the phycosphere of the blue-green algae was sequenced. The diffusive boundary layer immediately surrounding cyanobacterial cells or colonies (Ramanan *et al.* 2016), otherwise known as the phycosphere, is a resource-rich environment for bacterial colonizers such as sediminibacteria. Microalgae secrete 5–40% of their total photosynthate into the phycosphere, and of this mucilage, 30–90% can be utilized by heterotrophic bacteria (Kaplan and Bott 1982; Haack and McFeters 1982; Stevenson *et al.* 1996). The nutrients, metabolites, and signaling molecules that are exchanged between the bacteria and the microalgae in this microenvironment dictate the nature of their relationship which can be mutualistic, commensal, antagonistic, parasitic, or competitive. Bacteria and their association with cyanobacterial hosts from lakes and streams, particularly with respect to the phycosphere, is profoundly understudied. The genomes reported here of sediminibacterial residents in the phycosphere of 2 benthic freshwater cyanobacteria will shed light on the nature of the relationship between the organisms, the specificity of their interactions, and the effector molecules that trigger their affiliation. The availability of the genome sequence, moreover, promises to open new avenues for research.

## Materials and methods

### Samples and collection sites

The novel bacterium was isolated from laboratory cultures of 2 stream benthic cyanobacterial strains, Coccoid cyanobacterium_CKK01 and Filamentous cyanobacterium LYN-RS (hereon referred to using their strain names). Coccoid cyanobacterium_CKK01 was collected epiphytically on green alga *Cladophora* on August 30, 2018, from Escondido Creek, an urban perennial stream subject to intense agricultural runoff, increased salinity, and nutrients. Filamentous cyanobacterium LYN-RS was isolated from large visible filamentous mats on the bottom of a nearly completely dry intermittent creek in Anza Borrego Desert on December 12, 2019. Both samples consisting of large visible filamentous algae were collected by forceps, placed in plastic bags with water from the habitat, and transported in coolers to the California Algae Lab at CSU San Marcos for cyanobacteria isolation. Coccoid cyanobacterium_CKK01 were detected during the observation of *Cladophora* filaments with an Olympus SZ61 Stereomicroscope (Olympus America Inc., Center Valley, Pennsylvania). The cells of this coccoid cyanobacterium were grouped in colonies attached to the surface of *Cladophora*. Coccoid cyanobacterium_CKK01 cells were removed by fine forceps and initially cultured on a solid 1% BG-11 medium (Sigma-Aldrich, Inc., St. Louis, MO) until dispersed colonies developed and these were used for monoclonal strain isolation and growth in liquid BG-11. A single filament of Filamentous cyanobacterium LYN-RS was isolated directly in liquid BG-11. Both nonaxenic cyanobacterial strains grew for 50 days at 20–23°C with an irradiance of 80 mmol photons m^–^^2^ s^–^^2^ and a 12:12 h light:dark cycle^2^. The cyanobacterial cultures were grown until dense biofilm was formed to provide enough biomass for high molecular weight DNA extraction to obtain both cyanobacterial and bacterial genomes. Both cultures were also tested for production of cyanotoxins microcystins, anatoxins, saxitoxins, and cylindrospermopsins by liquid chromatography–tandem mass spectrometry as described in Conklin *et al.* (2020).

### Transmission electron microscopy

**Fig. 1.**
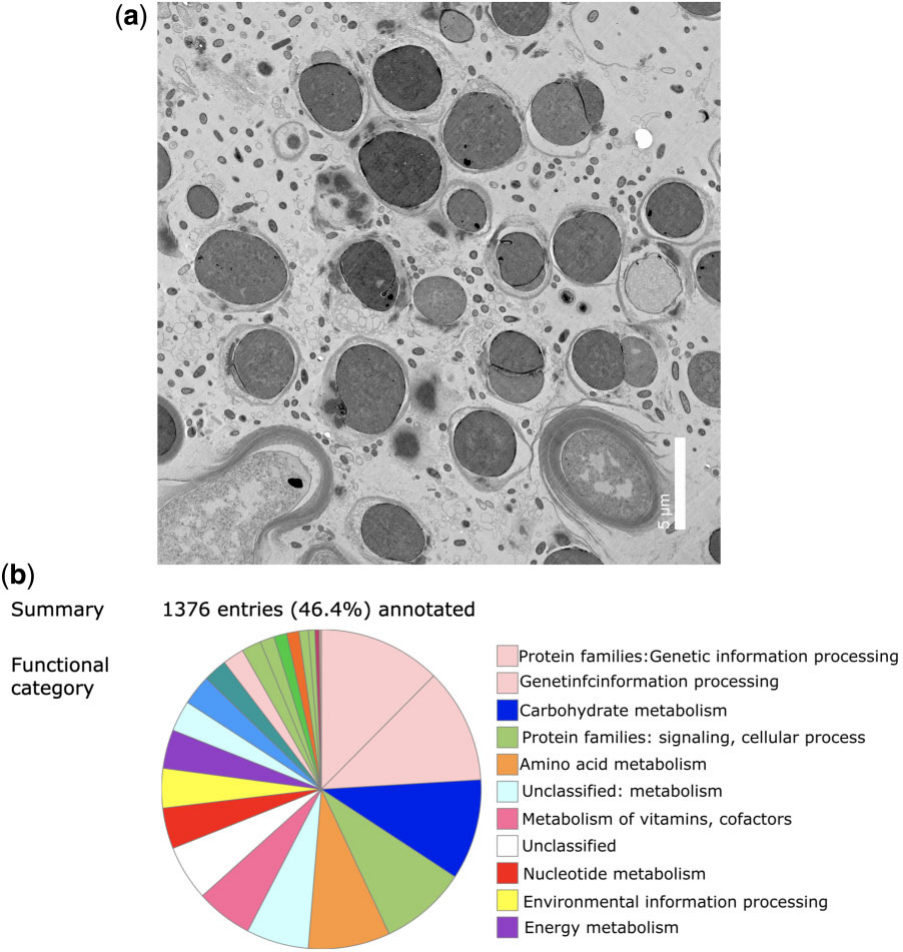
a) (L) TEM micrograph showing our novel *Sediminibacterium* sp. within the phycosphere of the cultured Coccoid cyanobacterium_CKK01. b) (R) KEGG functional classification of approximately 47% of all annotated genes.

Transmission electron microscopy (TEM, Fig. 1a) was performed with fresh cultured material from both cyanobacterial strains as described in Stancheva *et al.* (2019). Briefly, cells were fixed with 2% glutaraldehyde in 0.1 M cacodylate buffer and postfixed in 1% OsO4 in 0.1 M cacodylate buffer for 1 hr on ice. The colonies were dehydrated in graded series of ethanol (50–100%) while remaining on ice. They were then subjected to 1 wash with 100% ethanol and 2 washes with acetone (10 min each) and embedded with Durcupan. Sections were cut at 60 nm on a Leica UCT ultramicrotome and picked up on 300 mesh copper grids. Sections were post-stained with 2% uranyl acetate for 5 min and Sato’s lead stain for 1 min. Cells were viewed using FEI Tecnai Spirit G2 BioTWIN TEM and photographed with a bottom mount Eagle 4k (16 MP) camera (Hillsboro, OR) at the Department of Cellular and Molecular Medicine at University of California San Diego.

**Fig. 2.**
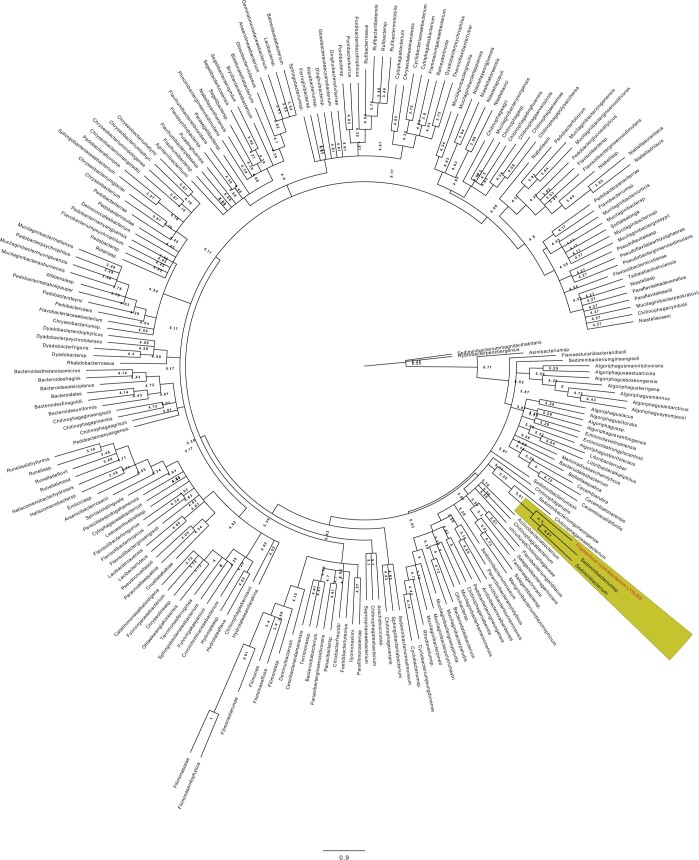
Phylogenomic reconstruction of the evolutionary history of our novel sediminibacterial strains (Coccoid cyanobacterium_CKK01 and Filamentous cyanobacterium LYN-RS) through consensus species tree reconstruction using 100 randomly selected single-copy gene trees. Both strains are sister to each other, and placed within a clade comprising other sediminibacterial strains.

**Fig. 3.**
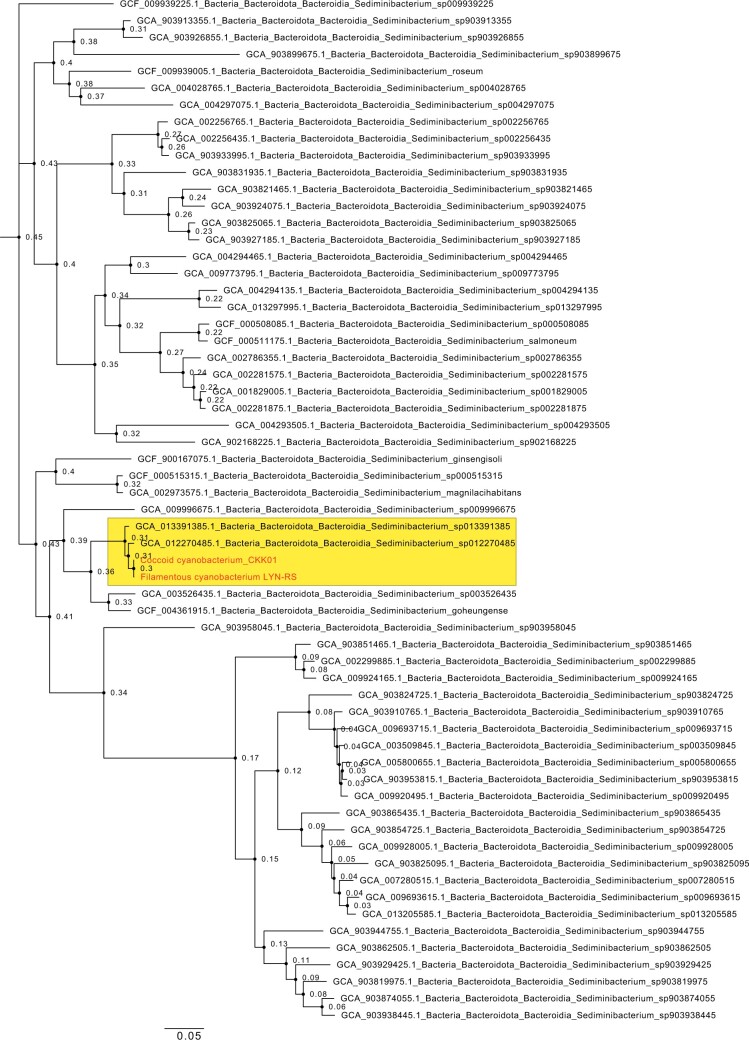
Phylogenomic reconstruction of the evolutionary history of our novel sediminibacterial strains (Coccoid cyanobacterium_CKK01 and Filamentous cyanobacterium LYN-RS) using GToTree, showing its monophyletic relationship amongst sediminibacterial strains GCA013391385 and GCA012270485. This reconstruction specifically utilized single-copy genes across all sediminibacterial genomes present in GenBank.

### DNA extraction and genome sequencing

High molecular weight genomic DNA was isolated using a standard CTAB chloroform extraction protocol followed by cesium chloride gradient centrifugation (Wilson 2001) from the whole culture containing both cyanobacterial host and the phycosphere sediminibacteria. The purity of the DNA was verified using absorbance ratios at A260/280 and A260/230 while quantity was determined by the absorbance at A260 on a nanodrop spectrophotometer. The integrity of the DNA was assessed using a 0.8% agarose gel. Sequencing libraries were prepared using the Transposase Enzyme Linked Long-read Sequencing (TELL-Seq) Whole Genome Sequencing (WGS) Library Prep Kit from Universal Sequencing (Carlsbad, CA). Briefly, 500 ng of genomic DNA was barcoded with 8 million TELL beads. After capture, barcoded DNA underwent 14 cycles of PCR amplification. The libraries were pooled and sequenced at the Scripps Research Institute (San Diego, CA) on an Illumina TC NextSeq 500/550 Mid Output Kit V2.5 (150 cycles) platform, which generated of 210 Gb of raw data for each of the 2 libraries. Demultiplexed raw reads were subjected to quality control using FastQC (FastQC 2016), and bases with a PHRED Q score of <30 were trimmed.

### Assembly

Raw sequence output was processed using the Tell-Read pipeline (Universal Sequencing, Carlsbad, CA); adaptor sequences were removed and BCL data were converted to linked read FASTQ files. Thereafter, the Tell-Link de novo assembly pipeline (Universal Sequencing) was applied. Barcode information was used to unravel complex structures in the de Bruijn assembly graphs at the global level. A local assembly process was then initiated to resolve the global graph by identifying sets of reads that share barcodes between 2 edges. This collection of reads was used to reconstruct de Bruijn graphs in local assemblies. To arrive at the best assembly, different combinations of k-mer sizes were tested whereby global k-mer sizes ranged from 45 to 55, and local k-mer sizes varied between 27 and 31. The optimal k-mer combination was determined to be 55 and 31 for the global and local assemblies, respectively.

### Assembly quality, annotation, and phylogeny

Quality and completeness of the assemblies were assessed using QUAST v. 5.0.2 (Gurevich *et al.* 2013) and BUSCO v. 5.3.2 (Seppey *et al.* 2019) against the odb_bacteria10 gene family database followed by automated gene calling and functional analyses using the NCBI Prokaryotic Genome Annotation Pipeline (PGAP; Tatusova *et al.* 2016) and KEGG (Kanehisa *et al.* 2008) using the BlastKOALA web interface (Kanehisa *et al.* 2016), querying against the genus_prokaryotes database. GapMind (Price *et al.* 2020) was then utilized to identify amino acid biosynthesis pathways in our PGAP-annotated genome. For phylogenetic analyses, all annotated protein-coding nucleotide sequences were used as queries against the nr database in NCBI using BLASTP (Altschul *et al.* 1990), and the top 50 hits were obtained as multiple sequence alignments with Mview (Brown *et al.* 1998). Thereon, we identified single-copy gene markers by (1) filtering out alignments with species that were only present in one of the queries and (2) retaining only alignments where a particular species hit was present in more than 90% of queries. RAxML v.8.0 (Stamatakis 2014) with the *PROTGAMMAAUTO* option was then used to generate single-copy gene trees from a random set of 100 single-copy genes. These gene trees were then pooled to generate a consensus species tree using ASTRAL v.5.1.1 (Mirarab *et al.* 2014). A BLASTN homology search was also performed to compare and establish the novelty of our genome against sister species of sediminibacteria identified in the ASTRAL species tree. To resolve the phylogenomic position of Coccoid cyanobacterium_CKK01 and Filamentous cyanobacterium LYN-RS within sediminibacteria, we also utilized GToTree (Lee 2019) against all available sediminibacterial genomes in GTDB (Parks *et al.* 2022). Briefly, we searched GTDB for “*Sediminibacterium*,” which produced a total of 65 accession hits. GToTree then automates (1) querying and downloading all genomes from GenBank, (2) translating the Coccoid cyanobacterium_CKK01 and Filamentous cyanobacterium LYN-RS in all open reading frames, (3) filtering all genomes for 90 single-copy genes against the Bacteroidetes HMM-gene set, retaining only single-copy genes present in at least 90% of all genomes, (4) performing multiple sequence alignments, followed by (5) phylogenetic reconstruction of the concatenated multiple sequence alignment. Furthermore, we also utilized GTDB-TK v.1.7.0 (Chaumeil *et al.* 2020) via the kbase.us web interface to place our genome assemblies among all available bacterial genomes in GTDB (Parks *et al.* 2020) using classification of bacterial Operational Taxonomic Units (OTUs).

## Results and discussion

Genome assemblies of sediminibacteria derived from the phycosphere of Coccoid cyanobacterium_CKK01 and Filamentous cyanobacterium LYN-RS revealed the presence of a single species of bacterium (99% Average Nucleotide Identity between assemblies). The most contiguous of the assemblies comprised a single contig of length 3.34 Mbps, with 43.06% GC, while the other assembly was split into several smaller contigs (Table 1). Both assembled genomes have been submitted to NCBI Project SAMN23222054 and are also accessible via the project’s GitHub page. The novelty of these genomes was established using genomic similarity with 28 other sediminibacterial genomes, with the highest BLASTN identity (81.46%) to NCBI Accession IDs: GCA_013391385.1, GCA_019264545.1, and GCA_019264645.1. Annotation using PGAP identified 3,009 genes, including 2,964 coding sequences, 2 pseudogenes, and 45 RNA sequences (3 rRNA, 39 tRNA, 3 ncRNA) with overall coding density of 752 genes per Mbps. The genome was identified to be 97% complete (BUSCO, bacterial-odb10—Table 1), with 1% of duplications, and 2% of missing families. Forty-seven percentage of all annotated genes were functionally classified using KEGG (Fig. 1b). Species tree reconstruction using ASTRAL placed our novel bacterium as sister to sediminibacteria, with hot-spring chitinobacteria within the same super-clade (Fig. 2). Further phylogenomic resolution using GToTree (Fig. 3) placed the sister sediminibacterial novel genomes of Coccoid cyanobacterium_CKK01 and Filamentous cyanobacterium LYN-RS strains as monophyletic with *Sediminibacterium_sp013391385* (GCA_013391385.1) and *Sediminibacterium_sp012270485* (GCA_012270485.1), and sister to the monophyly consisting of *Sediminibacterium_sp003526435* (GCA_003526435.1) and *Sediminibacterium goheungense* (GCF_004361915.1). These results were also recapitulated by our GTDB-Tk analyses, which place the new assemblies closest to GCA_013391385.1 (93.4% ANI), GCA_012270485.1 (94.16% ANI), *Sediminibacterium goheungense* (GCF_004361915.1, 79.28% ANI), *Sediminibacterium_sp003526435* (GCA_003526435.1, 78.75% ANI), and *Sediminibacterium_sp009996675* (GCA_009996675.1, 77.84% ANI).

Analyses of the genomic sequence indicates that, in the absence of a lactate dehydrogenase and the presence of genes encoding oxidase, superoxidase dismutase, and catalase, the novel *Sediminibacterium* is strictly aerobic like *Sediminibacterium salmoneum* (Kim *et al.* 2013). While complete pathways for glycolysis, the citric acid cycle, pentose phosphate shunt, beta-oxidation, and the electron transport chain are present, there is no support for the Entner–Douroroff pathway, or the metabolism of formate, methane, or sulfate reduction. In addition, unlike many of the other sediminibacterial species where menaquinone-7 is the major electron carrier (Kim *et al.* 2013; Kang *et al.* 2014; Song *et al.* 2017), genes encoding key enzymes for the biosynthesis of both menaquinones (*mqnA*, *mqnB*, *mqnC*, *mqnD*, *mqnE*, *mqnX*, *menI*, *ubiE*) and ubiquinones (*ubiA*, *ubiD*, *ubiE*, *ubiG*, *ubiH*, *ubiX*) are present in this strain, indicating that both may be functional. When GapMind (Price *et al.* 2020) was used to curate amino acid biosynthetic pathways, enzymes for the synthesis of nearly all 20 amino acids were identified with high confidence. Proline and serine pathways, however, could not be resolved, with enzymes for *N*-acetylornithine aminotransferase, *N*-acetylornithine deacetylase, and phosphoserine phosphatase either missing or highly divergent. Complete pathways, moreover, could not be identified when using BlastKOALA (Kanehisa *et al.* 2016), for the biosynthesis cobalmin, thiamin, biotin, and tetrafolate suggesting either the presence of variant pathways and enzymes or that this strain of *Sediminibacterium* is auxotrophic for these vitamins and cofactors. Notable among the 65 identified transporters were those for starch (*TonB*, *SusD*), maltose (*MalY*), futose (*fucP*), arabinose (*araE*), lipopolysaccharide (*lptF*, *lptG*, *lptB*), aquaglyceroporin (*GLPF*), nucleoside (*Yhhq*, *xapB*, *yhhQ*), and several different forms of iron (*ccmB*, *ccmC*, *feoA*, *VIT*, *TC.FEV.OM*).

**Table 1.** Genome assembly summary and completeness statistics from QUAST v.5.0.2 and BUSCO v.5.3.2 for both sediminibacterial assemblies derived from Coccoid cyanobacterium_*CKK*01 and Filamentous cyanobacterium LYN-RS.

Statistics	Coccoid cyanobacterium_CKK01	Filamentous cyanobacterium LYN-RS
Largest contig	2,242,242.0	3,342,270.0
Total length	5,062,689.0	5,117,762.0
Total length (≥0 bp)	5,062,689.0	5,117,762.0
Total length (≥1,000 bp)	4,415,391.0	4,276,438.0
N50	875,205.0	3,342,270.0
N75	2,734.0	2,470.0
L50	2.0	1.0
L75	134.0	154.0
GC (%)	42.12	43.06
#N’s	1,500	1,428
#N’s per 100 kbp	29.63	27.9
BUSCO groups searched against odb_bacteria10		
Complete BUSCOs (C)	120 (96.8%)	121 (97.6%)
Complete and single-copy BUSCOs (S)	119 (96%)	120 (96.8%)
Complete and duplicated BUSCOs (D)	1 (0.8%)	1 (0.8%)
Fragmented BUSCOs (F)	0 (0.0%)	1 (0.8%)
Missing BUSCOs (M)	4 (3.2%)	2 (1.6%)
Total BUSCO groups searched	124 (100%)	124 (100%)

Sediminibacteria are affiliated with microalgae in environmental samples and in laboratory cultures. For example, bacterial commensals have been described in the phycosphere of bloom-forming cyanobacterium *Microcystis aeruginosa* in lakes (Kim *et al.* 2019) and in the phycosphere of laboratory cultures of *Micrasterias* (Zygnematophyceae) (Krohn-Molt *et al.* 2013). Media for culturing cyanobacteria generally contains high concentrations of inorganic nutrients (N, P, Fe) but no added carbon sources, and hence bacteria must rely on the transport of organic matter either actively secreted by the microalgae or passively released during cell death or phage lysis. Exoenzymes including alkaline phosphatase, ß-glucosidase, glucosaminidase, and aminopeptidases encoded in the genome of the *Sediminibacterium* colonizers here may provide access to these macromolecules in batch cultures, but could also do so in freshwater streams and lakes, where the concentration of organic matter in the phycosphere of microalgae is often elevated compared to that of the surrounding waters (Seymour *et al.* 2017).

The ability to reach the phycoshpere enables sedminibacteria to escape nutrient starvation in the bulk water, while the ability to detect molecules and move toward them provides a competitive advantage for this motile and chemotactic bacteria over nonmotile nonchemotactic species. The lack of most commonly produced freshwater cyanotoxins in the phycosphere of both hosts may facilitate the penetrations of sediminibacteria. The importance of motility and chemotaxis in the ecology of Sediminbacteria is supported by genomic evidence for many othologous genes involved in chemotaxis, motility, attachment, and quorum sensing discovered in our annotation. Chemotaxis genes include those that encode a histidine kinase (*CheA*) and an adaptor protein (*CheW*) that assemble into signaling complexes; a response regulator that interacts with the flagellar motor (CheY); and 2 methyltransferases (*CheB* and *CheR*). Flagellar structural and the motor component genes (*flgN*, *flgG*, *flgJ*, *motB*, and *motA*/*tolQ*/*exbB*) are also detected, in addition to those that encode proteins conferring rapid gliding motility over surfaces (*gldC*, *gldG*, *gldH*, *sprA*, *sprF*, *sprT*) and elements of the type IX secretion system T9SS. Together these genes likely enable sediminibacteria to move toward favorable environments rich in compatible hosts and/or dissolved organic carbon, and away from detrimental conditions. Genomic evidence also suggests that *Sediminibacterium* has evolved elaborate biofilm and quorum sensing mechanisms for attaching to and colonizing surfaces. More than 30 biofilm formation and quorum sensing genes involved in processes such as signal transduction and secretion (*uvrY*, *gacA*, *varA*, *secE*, *SecG*, *SecY*, *SecDF*, *ftsY*, *yajC*, *yidC*, *spoIIIJ*, *OXA1*, *ccfA*), transcription (*crp/fnr*, *ribD*, *oxyR*/*Lsy R*), carbohydrate metabolism (*pslH*, *glgP*, *glgA*, *glgC*, *pgaC*, *icaA*), surface adhesion proteins (*BapA*), and multidrug resistance (*oprM*, *emhC*, *ttgC*, *cusC*, *adeK*, *smeF*, *mtrE*, *cmeC*, *gesC*), are present in these genomes.

The extent to which freshwater algal–bacteria interactions are specific and/or how the bacterial colonizers are selected by, or choose their microalgal hosts is not well understood. Mutualism whereby bacteria provide essential vitamins and phytohormones (Croft *et al.* 2005; Grant *et al.* 2014), antimicrobial defense (Seyedsayamdost *et al.* 2011; Seymour *et al.* 2017), recycled nutrients (Christie-Oleza *et al.* 2017), and/or protection from oxidative stress (Hünken *et al.* 2008; Morris *et al.* 2011) in exchange for nutrients and shelter has been documented for several bacterial microalgal affiliations. While it is tempting to speculate that a mutualistic relationship exists between the sediminibacteria and the cyanobacteria in this study, experimental evidence of this nature awaits further investigation. Although genes encompassing the complete pathways for the synthesis of vitamins (B12, thiamin, and biotin) often supplied by cyanobacterial symbionts were not identified, genomic evidence suggests that the *Sediminibacterium* may offer protection against colonization of opportunistic bacteria by producing bacteriocin and toxoflavin. In return, the cyanobacteria provide a stable microhabitat and nutrient supply for the bacteria.

## Data availability

The genome will be made publicly available via NCBI BioSample: SAMN23222054. All assemblies, scripts utilized in assembly, annotation, and phylogenetic reconstruction can be accessed at www.github.com/arunsethuraman/sediminibacterium.
